# Supplementary material for: The genome sequence of the Holly Tortrix, Rhopobota naevana (Hubner, 1817) (Lepidoptera: Tortricidae)
Source: Wellcome Open Res. 2026 Jun 27;11:400. [Version 1] doi: 10.12688/wellcomeopenres.26882.1 (PMC13560743; doi:10.12688/wellcomeopenres.26882.1)
Supplement: Peer review history

# Reviewer report: Reviewer response for version 1

**DOI:** 10.21956/wellcomeopenres.29601.r162916

**Published:** 2026-08-18

**Authors:** Nicola J Nadeau

**Affiliations:** 1 The University of Sheffield, Sheffield, UK

This note reports the reference genome generated for the moth *Rhopobota naevana. *Moths are ecologically important as prey for many larger species, pollinators as adults and pest herbivores as larvae. The note gives useful background information on the ecology of this species. 

 The data note gives a detailed description of the methods, and a clear report of the data and assembly generated. The work is thorough and conducted to the recognized standards in the field. The assemblies and raw data are mostly available in an accessible form on ENA. However, data for the alternate haplotype (component project PRJEB91880) seems to be missing. The project also seems to include an assembly of the Wolbachia endosymbiont. While a full description of this assembly is presumably going to be published separately, it might be worth including a mention of this in the current note.

 I have a couple of other minor suggestions:

 "The MitoHiFi reference was the mitochondrial genome of *Epinotia rubiginosana" - *It might be useful to elaborate on the taxonomic relationship of *Epinotia rubiginosana *to *Rhopobota naevana *or say something about how similar their Mito genomes are *.*

 " Z chromosomes were identified based on BUSCO gene painting with ancestral Merian elements" - It would be useful to explain what Merian elements are for those who are not familiar. I also wonder if this chromosome should really be annotated as the Z without any other supporting information (e.g. coverage in females). It would be more conservative to simply give it a number at this stage.

Are sufficient details of methods and materials provided to allow replication by others?

Yes

Is the rationale for creating the dataset(s) clearly described?

Yes

Are the datasets clearly presented in a useable and accessible format?

Yes

Are the protocols appropriate and is the work technically sound?

Yes

Reviewer Expertise:

evolutionary genetics/genomics, lepidoptera

I confirm that I have read this submission and believe that I have an appropriate level of expertise to confirm that it is of an acceptable scientific standard.

# Reviewer report: Reviewer response for version 1

**DOI:** 10.21956/wellcomeopenres.29601.r160588

**Published:** 2026-07-28

**Authors:** Sivasankaran Kuppusamy

**Affiliations:** 1 Loyola College, Chennai, tamilnadu, India

The authors have successfully generated a chromosome-level genome assembly for *Rhopobota naevana* (Hubner, 1817), with a total genome size of 581. 80 megabases. They have employed appropriate methodologies for High Molecular Weight DNA extraction, DNA quantification and SMRT library preparation and sequencing using Illumina and PacBio platforms. The genome assembly and annotations were carried out using well established bioinformatic software and pipelines. Overall, the genome analysis is technically sound, and the methodologies adopted are appropriate for producing a reliable chromosome-level genome assembly. 

**Comments on the manuscript**

 Authors have generated 132.28 Gb of high-quality sequencing data through Illumina and PacBio pipelines. However, authors have not reported the protein-coding genes non-coding genes and gene transcripts. The details can be included in the genome assembly table.  

 The Longest scaffold (Mb), GC content (%) and BUCSCO completeness (%) are the important assembly metrics and can be included in the genome assembly table

 Overall, the research article was well prepared and the manuscript meets the necessary scientific standard and is suitable for indexing

Are sufficient details of methods and materials provided to allow replication by others?

Yes

Is the rationale for creating the dataset(s) clearly described?

Yes

Are the datasets clearly presented in a useable and accessible format?

Yes

Are the protocols appropriate and is the work technically sound?

Yes

Reviewer Expertise:

Phylogenetic analysis of superfamily Noctuoidea moths using mitogenome analysis

I confirm that I have read this submission and believe that I have an appropriate level of expertise to confirm that it is of an acceptable scientific standard.
